# Supplementary material for: Diagnostic Accuracy of Magnetic Resonance Imaging in the Detection of Type and Location of Meniscus Tears: Comparison with Arthroscopic Findings
Source: J Clin Med. 2021 Feb 5;10(4):606. doi: 10.3390/jcm10040606 (PMC7914628; doi:10.3390/jcm10040606)
Supplement: Supplementary file 1 [file jcm-10-00606-s001.pdf]

Table S1. Type of medial meniscus tear in ACL injured patients.

A/S<br>MRI	Longitudinal	Horizontal	Radial	Vertical Flap	Horizontal Flap	Complex	No tear	Total
Longitudinal	27	0	0	0	0	3	8	38
Horizontal	0	2	0	0	0	0	7	9
Radial	4	1	2	0	0	5	5	17
Vertical Flap	1	0	0	0	0	0	0	1
Horizontal Flap	1	0	0	0	0	0	0	1
Complex	13	2	0	0	0	26	3	44
No tear	10	1	1	0	0	0	70	82
Total	56	6	3	0	0	34	93	192

Kappa :0.511 p=0.045.

Table S2. Location of medial meniscus tears in ACL injured patients.

A/S<br>MRI	Anterior horn	Body	Posterior horn	More than 2 compartment	No tear	Total
**Anterior horn**	0	0	0	1	2	3
**Body**	0	3	0	0	0	3
**Posterior horn**	0	1	35	8	14	58
**More than 2 compartment**	1	2	11	25	8	47
**No tear**	1	0	8	3	69	81
**Total**	2	6	54	37	93	192

Kappa: 0.519 0.048.

Table S3. Type of lateral meniscus tear in ACL injured patients.

A/S<br>MRI	Longitudinal	Horizontal	Radial	Vertical Flap	Horizontal Flap	Complex	No tear	Total
Longitudinal	11	2	1	0	0	1	2	17
Horizontal	1	1	1	1	0	4	3	11
Radial	0	0	14	0	0	4	4	22
Vertical Flap	1	0	0	0	0	0	2	3
Horizontal Flap	0	0	0	0	0	0	1	1
Complex	7	2	0	1	0	16	6	32
No tear	15	2	8	0	0	8	73	106
Total	35	7	24	2	0	33	91	192

Kappa: 0.408 0.048.

Table S4. LM tear involved location in ACL injured patients.

A/S<br>MRI	Anterior horn	Body	Posterior horn	More than 2 compartment	No tear	Total
Anterior horn	4	1	0	3	2	10
Body	0	4	0	2	3	9
Posterior horn	0	1	23	5	9	38
More than 2 compartment	0	3	6	15	4	28
No tear	1	4	24	5	73	107
Total	5	13	53	30	91	192

Kappa 9: 0.396 0.05.

Table S5. Type of medial meniscus tear in ACL intact patients.

A/S \ MRI	Longitudinal	Horizontal	Radial	Vertical Flap	Horizontal Flap	Complex	No tear	Total
Longitudinal	14	2	0	0	0	2	2	20
Horizontal	1	18	1	0	0	23	18	61
Radial	3	0	5	0	0	7	7	22
Vertical Flap	0	0	0	0	0	0	0	0
Horizontal Flap	0	0	0	0	0	0	1	1
Complex	4	5	3	1	0	64	11	88
No tear	2	1	4	0	0	2	150	159
Total	24	26	13	1	0	98	189	351

Kappa :0.573 p=0.033.

Table S6. MM tear involved location in ACL intact patients.

A/S \ MRI	Anterior horn	Body	Posterior horn	More than 2 compartment	No tear	Total
Anterior horn	0	0	0	1	2	3
Body	0	4	2	2	3	11
Posterior horn	0	1	43	5	29	78
More than 2 compartment	0	9	23	61	8	101
No tear	3	5	1	2	147	158
Total	3	19	69	71	189	351

Kappa: 0.548 0.033.

Table S7. Type of lateral meniscus tear in ACL intact patients.

A/S \ MRI	Longitudinal	Horizontal	Radial	Vertical Flap	Horizontal Flap	Complex	No tear	Total
Longitudinal	34	2	1	0	0	6	0	43
Horizontal	1	12	2	0	0	11	5	31
Radial	5	3	15	0	0	10	11	44
Vertical Flap	0	0	0	3	0	0	1	4
Horizontal Flap	0	0	0	0	0	0	0	0
Complex	8	5	6	1	0	26	6	52
No tear	7	3	4	0	0	5	158	177
Total	55	25	28	4	0	58	181	351

Kappa: 0.568 0.032.

Table S8. LM tear involved location in ACL intact patients.

A/S \ MRI	Anterior horn	Body	Posterior horn	More than 2 compartment	No tear	Total
Anterior horn	5	9	0	5	2	23
Body	0	26	3	2	3	40
Posterior horn	1	2	12	9	9	30
More than 2 compartment	2	12	4	59	4	81
No tear	1	6	6	6	158	177
Total	9	55	25	81	181	351

Kappa: 0.582 0.032.
